# Impact of high altitude on composition and functional profiling of oral microbiome in Indian male population

**DOI:** 10.1038/s41598-023-30963-8

**Published:** 2023-03-10

**Authors:** Manisha Kumari, Brij Bhushan, Malleswara Rao Eslavath, Ashish Kumar Srivastava, Ramesh Chand Meena, Rajeev Varshney, Lilly Ganju

**Affiliations:** 1 Pathophysiology and Disruptive Technologies, Defence Institute of Physiology and Allied Sciences (DIPAS), Defence Research and Development Organisation (DRDO), Lucknow Road, Timarpur, Delhi 110054 India; 2 Department of Pediatrics Newborn Medicine, Brigham and Women’s Hospital, Harvard Medical School Boston, 02115 Massachusetts, United States

**Keywords:** Applied microbiology, Bacteria, Microbial communities, Computational biology and bioinformatics

## Abstract

The oral cavity of human contains bacteria that are critical for maintaining the homeostasis of the body. External stressors such as high altitude (HA) and low oxygen affect the human gut, skin and oral microbiome. However, compared to the human gut and skin microbiome, studies demonstrating the impact of altitude on human oral microbiota are currently scarce. Alterations in the oral microbiome have been reported to be associated with various periodontal diseases. In light of the increased occurrence of HA oral health related problems, the effect of HA on the oral salivary microbiome was investigated. We conducted a pilot study in 16 male subjects at two different heights i.e., H1 (210 m) and H2 (4420 m). Total of 31 saliva samples,16 at H1 and 15 at H2 were analyzed by utilizing the 16S rRNA high-throughput sequencing, to explore the relationship between the HA environment and salivary microbiota. The preliminary results suggesting that, the most abundant microbiome at the phylum level are: *Firmicutes*, *Bacteroidetes, Proteobacteria,* and *Actinobacteria.* Interestingly, 11 genera were identified at the both heights with different relative abundances. In addition, the salivary microbiome was more diverse at H1 compared to H2 as demonstrated by decreased alpha diversity. Further, predicted functional results indicate that microbial metabolic profiles significantly decreased at H2 as compared to H1, including two major metabolic pathways involving carbohydrates, and amino acids. Our findings show that HA induces shifts in the composition and structure of human oral microbiota which can affect host health homeostasis.

## Introduction

Human oral microbiota is the second most complex and diverse microbial community after gut, inhabited principally by *Firmicutes*, *Bacteroidetes*, *Proteobacteria*, *Actinobacteria*, *Spirochaetes* and *Fusobacteria*^1^. As per the Human Oral Microbiome Database more than 1100 different taxa are reported^2^, out of which genera *Streptococcus*, *Veillonella*, *Neisseria*, and *Actinomyces* are associated with the core microbiome, shared by most healthy individuals^3^. Presence of various niches inside the oral cavity and the nasopharyngeal areas provide suitable environment for microorganisms to grow^2^.

Composition of oral microbiota is influenced by host genetics^4^, geography^5^, diet^6^, age^7^, and environment^8^, suggesting that periodontal health or disease depends on the interface between the host and the microbial community as a whole. To be specific, oral microbial diversity is a strong indicator of oral health and overall human health. Dysbiosis, or an imbalance in the oral microbiome, has been related to various local and systemic human disorders, such as dental caries, obesity, diabetes, and cardiovascular disease^2,9–11^.

Studies demonstrating the impact of altitude on human oral microbiota are currently scarce. However, much recent evidence accumulated from Tibetan plateau shows that diversity in oral microbiota gets altered living at different altitudes and the ecological mechanisms associated with it respond differently as compared to low altitude natives. Recent studies on animal models exposed to chronic hypoxia showed increased risk of periodontitis development due to increased oxidative stress and inflammatory parameters in sub-mandibular glands^12,13^. A study conducted at an elevation of 3550 m reported a prevalence of dental problems such as gingival bleeding, dental pain, lost fillings and dental fractures in 23.2% of trekkers^14^. A significant decrease in salivary flow^15^ has also been reported during prolonged stay at HA areas^16^ which is known to increase the risk of caries^17^. Studies conducted at Tibetan plateau showed that oral microbiota is much more diverse at low altitude as compared to the HA Zhang population (living at an altitude of 3000–4000 m)^18,19^. The study also found high abundance of *Porphyromonas gingivalis* in people living at HA^19^. *Porphyromonas gingivalis* is closely related to the occurrence of periodontal diseases and is one of the main microbes detected in the saliva of periodontitis patients^20^. Studies have also established a po+sitive correlation between ecosystem stability and species diversity. One recent study at HA area of Qinghai-Tibet plateau (average altitude of 4000 m) has shown that alpha diversity decreases with altitude^21^, which might be responsible for increased occurrence of dental caries at HA (above 3500 m). The study also revealed an increased relative abundance of *Prevotella*, many species of which are prominent periodontal-pathogen. Understanding what constitutes microbial communities in oral cavity, is crucial as human mouth, the portal of entry to both the gastrointestinal (GI) and respiratory tract is in direct contact with the external environment and hence external environment plays a vital role in framing the oral microbiome.

In this study we assessed changes in oral microbiota composition in Indian male subjects of the same ethnicity who ascended to extreme altitude. To understand this, we used high-throughput 16S ribosomal RNA (rRNA) gene amplicon sequencing to characterize oral microbial diversity. It is already established that oral microbiota shares a close and intricate relationship with various health problems, the primary objective of this pilot study was to investigate the impact of HA on oral microbiota composition and functional prediction from healthy individuals ascending to HA.

## Materials and methods

### Subject recruitment

From a group of HA sojourners who had not ascended higher than 3000 m in the previous six months, sixteen participants of North Indian origin were selected. All the participants were males in the age group of 22–55 years, had normal weight (BMI = 20–24 kg/m2) and undergone thorough medical and psychological examinations for any diseases to ensure a healthy population. On the first day of examination, information on medication status was obtained by questionnaire and interview. No oral diseases, no recent antibiotic usage (within three months), and no eating or chewing gum two hours prior to sample collection were the inclusion criteria. Due to the harsh extreme environmental condition and logistic challenges, there was no enough choice of food items. The only food available was what the sojourners carried with themselves and hence everyone consumed similar type of food.

### Ethical statement

All participants understood the nature of the study and gave their written informed consent. Ethics approval was obtained from Research Ethics Committee of Defence Institute Physiology and Allied Sciences. All other study protocols were in accordance with Helsinki’s approved guidelines.

### Sample collection

Approximately 2 ml of passive saliva samples were collected in sterile vials between 7 and 9 am in the morning. All subjects were requested to refrain from drinking, eating, and oral hygiene activities (including rinsing with mouthwash) for at least 1 h before sample collection. The first sample collection was performed at sea level H1 (210 m) and second and final at H2 (4420 m) after staying for 6 months. Total of 31 saliva samples from 16 subjects at H1 and 15 subjects at H2 were analyzed for oral microbiota composition. The samples were placed on ice, and a protease inhibitor cocktail was added at a ratio of 100 ml per ml of saliva. Immediately after the addition of protease inhibitor, samples were frozen at −22 °C without culturing and finally transferred to −80 °C freezer till further processing.

### Sample preparation and DNA extraction

Two ml saliva collected in sterile vial was diluted with 4 ml PBS and centrifuged at 1800×g for 5 min. Genomic DNA was isolated from the pellet using the QIAamp DNA Mini Kit (Qiagen, Hilden, Germany). The quantity and quality of isolated DNA were measured using Nano Drop ND-1000 spectrophotometer (Thermo Fisher Scientific, Waltham, MA) and agarose gel electrophoresis (BioRad, USA), respectively. DNA extracted from samples were normalized and then stored at −20 °C until further use.

### Amplification of V3-V4 region of 16S rRNA, library preparation and sequencing

16S rRNA sequencing was conducted on Illumina MiSeq platform. To amplify and sequence the V3-V4 hyper-variable regions of the 16S rRNA gene, the 341F and 805R universal primers were used targeting a region of approximately 464 bp encompassing variability^22^.

The V3–V4 primer and the adapter details are as mentioned below.

V3- Illumina_16S_341F

5′-TCGTCGGCAGCGTCAGATGTGTATAAGAGACAGCCTACGGGNGGCWGCAG

V4-Illumina_16S_805R

5′GTCTCGTGGGCTCGGAGATGTGTATAAGAGACAGGACTACHVGGGTATCTAATCC

In accordance with the Human Microbiome Project (USA)^23^, this region provides sample information for the categorization and identification of microbial communities from specimens related with human microbiome investigations. The final amplified amplicon libraries were purified using AMPure XP beads (Beckman Coulter Genomics, Denver, MA, USA) and the size of the amplicon library wereassessed on the bioanalyser (Agilent technologies, USA). The library quantification kit for Illumina (Kapa Biosciences, Woburn, MA, USA) was used to assess the quantity of the amplicon. Paired-end (250 bp) sequencing was carried out at the Illumina MiSeq platform.

### Bioinformatic analysis of 16S rRNA gene amplicon sequences

Quality of the raw data was checked using FastQC software. Raw paired-end Illumina reads were trimmed using fastx toolkit (version 0.0.13). R1 and R2 paired-end reads were assembled, using PANDAseq and average read of ~ 464 base pairs was generated^24^. Sequencing of saliva samples for oral microbiota generated approximately a total of 1973 MB data for 31 saliva samples (approximately 63.6 MB data/sample) (Table S1). QC stats of the data have been shown in Table S2. Sequences were grouped into OTUs on the basis of similarity to known bacterial sequences (at 97% sequence similarity cut-off) available in the Greengenes databases (version 13.8; https://greengenes.secondgenome.com/)^25^ using QIIME 1.9.1^26^. Unmatched sequences were further clustered de novo based on pair-wise sequence identity. CSS normalization was applied to correct biases. Statistical analyses were executed using the Microbiome Analyst pipeline^27^.

### Functional profiling

High-quality sequencing data and sample information was used to predict the functional profiling of microbiome samples, using PICRUSt (version1.1.3)^28^ pipeline followed by STAMP (version 2.1.3) software to calculate functionally differential KEGG Pathways (www.kegg.jp/kegg/kegg1.html) between H1 and H2 group using Welch's test.

### Statistical analysis

31 Saliva samples (16 at H1 and 15 at H2) were analyzed by 16S rRNA sequencing. The alpha diversity was calculated by T-test and determined by Chao1, Shannon and Simpson’s. Beta diversity was determined by PERMANOVA (Permutational multivariate analysis of variance) and PCoA plots based on Bray–curtis dissimilarity distance were plotted (D-05 Unifrac for robust trade-offs between rare and abundant lineages). PERMANOVA was applied to identify statistical significance of beta diversity between groups (at 5% p value significance at phylum and genus level). Significantly differential microbiota at Phylum and genera level were mined using the EdgeR package of the language R and then visualized with a volcano plot. Multiple testing was corrected with FDR correction of the p-value at a 5% threshold.

## Results

Present study demonstrates the sequencing of V3-V4 regions of 16S rRNA from 31 saliva samples. In total, 1,881,630 sequences were obtained from the 31 samples, with an average sequence length of 251 bp (Table S1). The rarefaction curve of all samples calculated had reached a plateau, suggesting that the sequencing was sufficient to represent its true diversity (Fig. S1). Cumulative sum scaling (CSS) normalized relative abundance was calculated at the genus and phylum level (Tables S5 and S6). The mean sequence length was 251 base pair. The clustering of qualified sequences at 97% identity resulted in 1,292 OTUs (Table S7). The analysis was performed using QIIME1.9.1, PICRUSt 1.1.4 and Microbiome analyst (https://www.microbiomeanalyst.ca/), rarefaction curve of all samples reached a plateau, suggesting that the sequencing was deep enough at species level (Fig. S1).

### Oral microbiota composition

**Figure 1:**
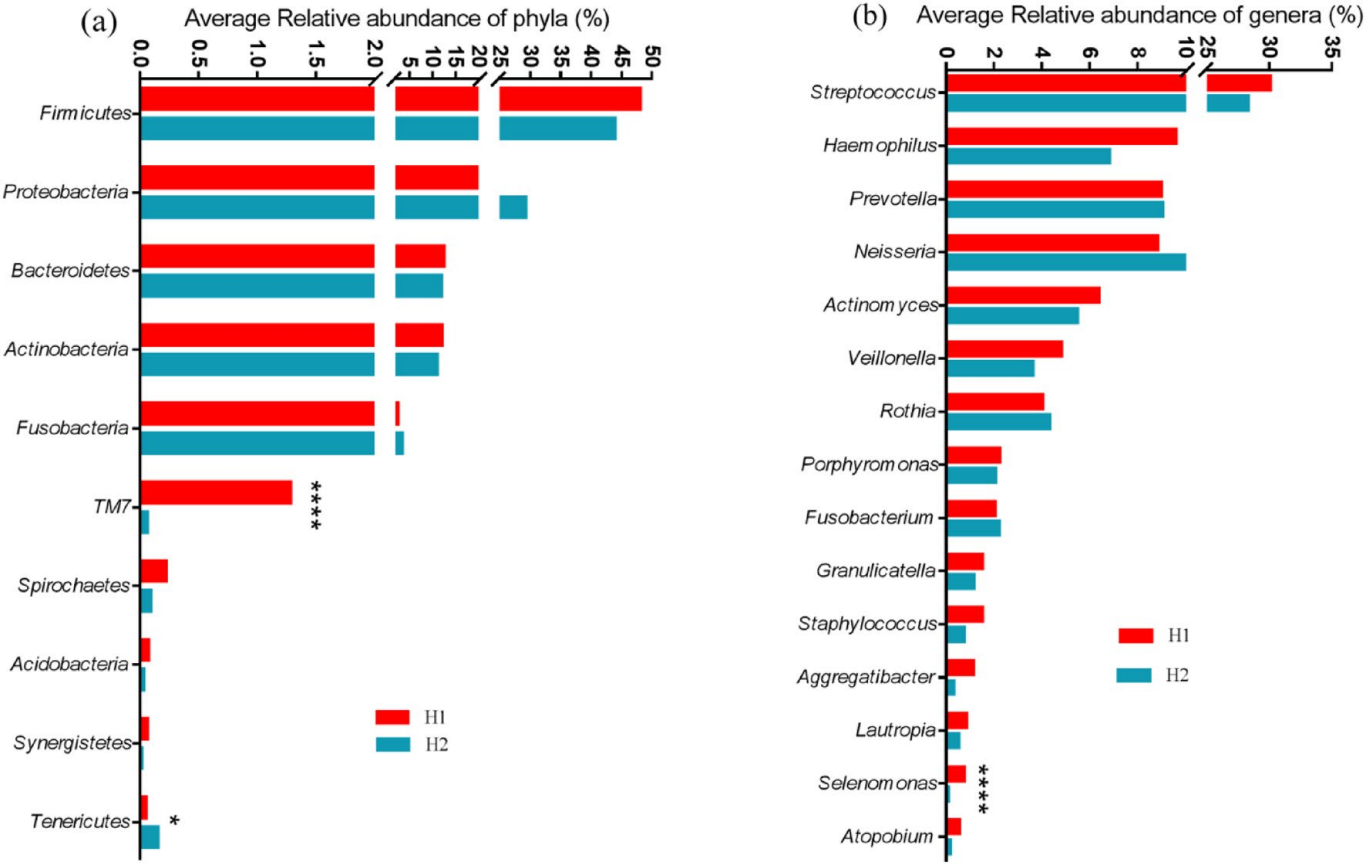
16S sequencing analysis of the variation in the oral microbiome at H1 and H2. (**a**) Bar chart depicts average relative abundance of the bacterial taxonomic hits at the phylum level in the saliva samples at H1 and H2 (**, P < 0.01, ****, P < 0.0001. For *TM7*, p = 0.0000002; for *Tenericutes*, p = 0.010). (**b**) Bar chart depicts average relative abundance of the bacterial taxonomic hits at the genera level in the saliva samples at H1 and H2 (****, P < 0.0001. For *Selenomonas*, p = 0.00002).

A total of 31 phyla, 59 classes, 95 orders, 136 families, 146 genera, and 48 species were detected from all the 31 samples. Microbial community, analyzed by 16S rRNA sequencing, at phylum level (Fig. 1a and b, and Tables S5) depicted that oral microbiota at H1 was dominated by *Firmicutes* (48%), followed by *Proteobacteria* (23%), *Bacteroidetes* (12%) and *Actinobacteria* (12%). After staying at H2 for six months there was a marginal decrease in *Firmicutes* (43%), *Bacteroidetes* (11%) and *Actinobacteria* (11%) and an increase in *Proteobacteria* (29%) (Fig. 1a), however, none of the changes reached significant. Out of the top 13 phylum (with relative abundance 0.1%), *TM7* (p < 0.0001, FDR q < 0.0001) and *Tenericutes* (p < 0.05, FDR q < 0.05) showed a significant alteration in their abundance at H2 (Fig. 1a).

At H1 time point, the most prevalent genera were *Streptococcus* (30.06%), *Haemophilus* (9.58%), *Prevotella* (8.96%), *Neisseria* (8.83%), *Actinomyces* (6.35%), *Veillonella* (4.79%), *Rothia* (4.01%), *Porphyromonas* (2.22%), *Fusobacterium* (2.03%), *Granulicatella* (1.49%), *Staphylococcus* (1.49%), *Aggregatibacter* (1.13%) and others with relative abundance less than 1% (Fig. 1b and Table S6). The predominant bacteria were largely consistent at both the heights, with different average relative abundances at H1 and H2. After stay at H2, the average relative abundance of *Streptococcus* decreased to 28.25%, followed by *Neisseria* (10.29%), *Prevotella* (9.04%), *Rothia* (4.30%) and *Fusobacterium* (2.18%) which increased but could not reach significant. On the contrary, genera *Haemophilus* (6.79%), *Actinomyces* (5.46%), *Veillonella* (3.62%), *Porphyromonas* (2.06%), *Granulicatella* (1.16%), *Staphylococcus* (0.75%), and *Aggregatibacter* (0.31%) were reduced. Comparing relative abundance of the 20 richest genera between two heights, a significant higher abundance of *Selenomonas* was observed at H1 as compared to H2 (p < 0.0001, FDR q < 0.0001) (Fig. 1b).

### Variations in oral microbiota diversity with altitude

**Figure 2:**
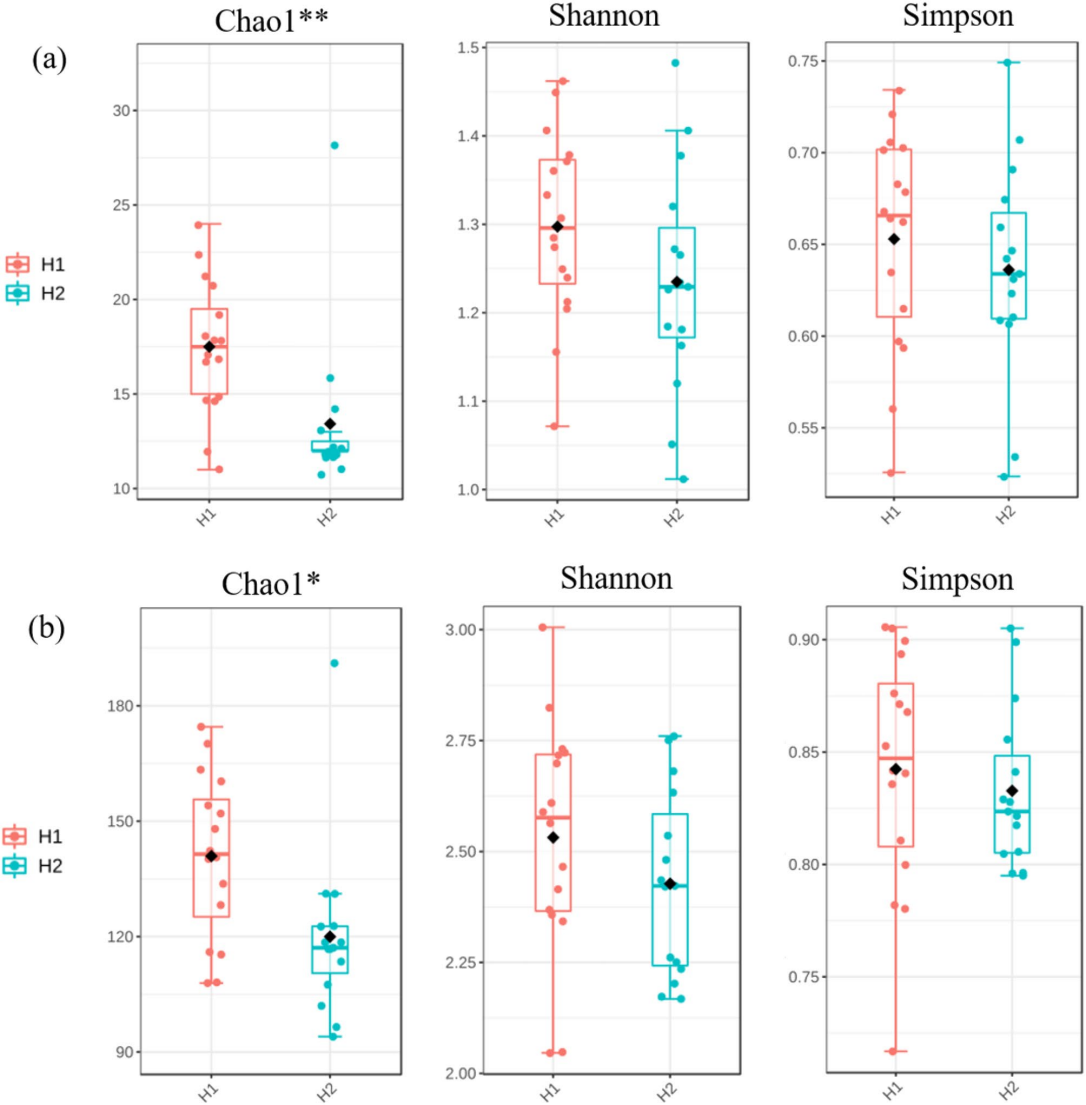
Comparison of alpha diversity between H1 and H2 at phylum and genus level. (**a**) Boxplots showing the differences in the alpha diversity indices (Chao 1, Shannon and Simpson) at phylum level at H1 and H2. (**b**) Boxplots showing the differences in the alpha diversity indices (Chao 1, Shannon and Simpson) at genus level at H1 and H2.

To understand the structural aspects of the microbial community various bacterial diversity metrices were employed. Three indices (Chao1, Shannon Index and Simpson), were employed to estimate the alpha diversity at different altitudes. A significant change was observed between H1 and H2 after the stay, according to Chao1 index at phylum and genus level (p = 0.007 and 0.012 respectively). On the other hand, neither the Shannon nor Simpson diversity indices reflected any significant difference (Fig. 2a, b, and Table S3).

**Figure 3:**
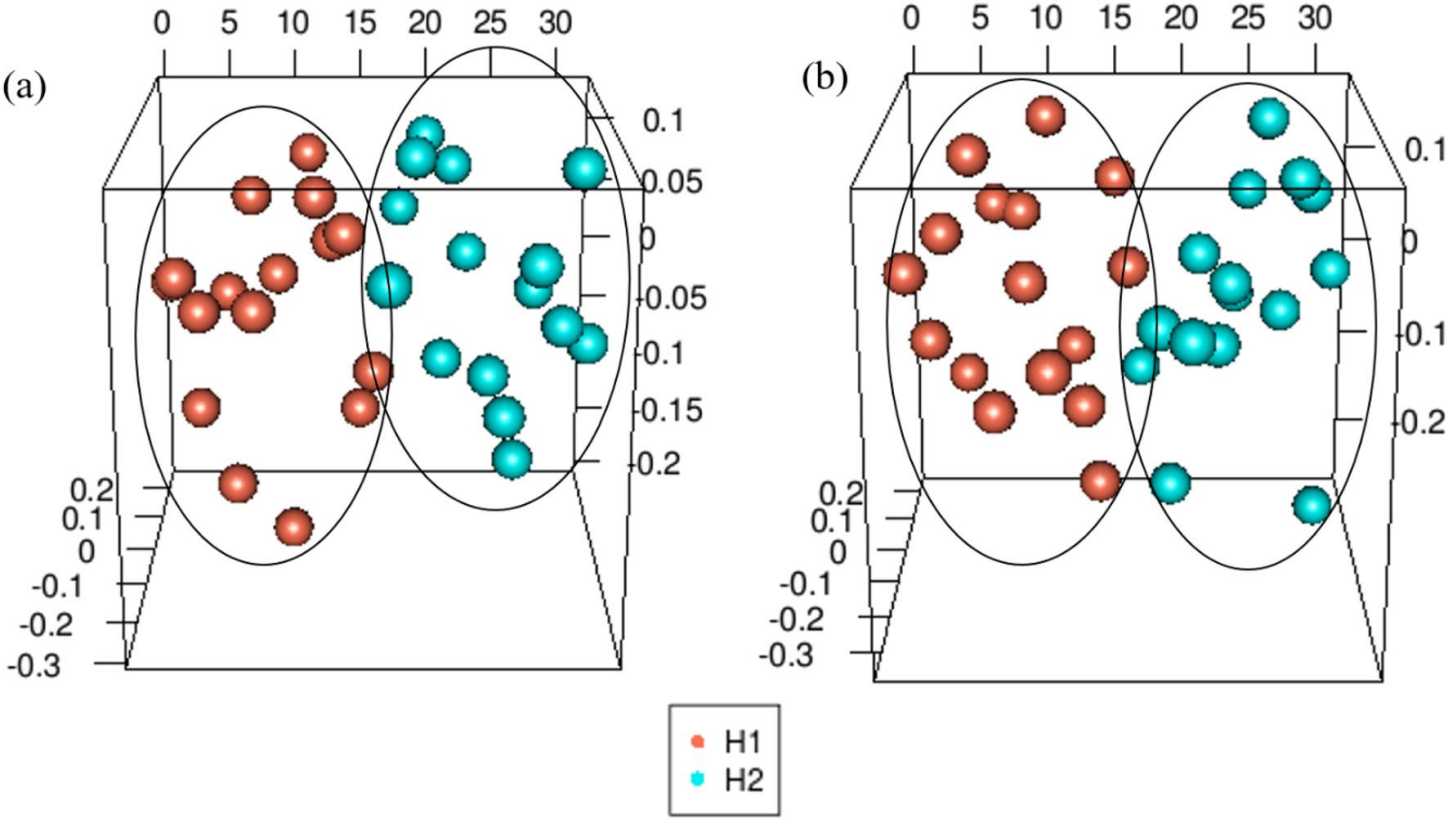
Principal Coordinate analysis (PCoA) on the distance matrix of Bray–curtis at H1 and H2 from 16S rRNA. PCoA of the oral microbiome at H1 and H2 at phylum (**a**) and genera level (**b**).

Beta diversity analysis was performed to assess the composition of the microbial communities between samples from the two heights. Based on the Bray–Curtis distances of the 16S ribosomal DNA sequencing profiles at phylum and genus level, PERMANOVA analysis and PCoA plot was generated. Results demonstrated separate clusters, suggesting some differences in the communities (significant differential distribution of oral microbiota at p = 0.05), between altitudes H1 and H2 (Fig. 3a, b, and Table S4).

### Predicted functional profiling of oral microbiota at H1 and H2

To evaluate the effect of altitude on oral microbiota, high-quality reads from all samples were assembled and annotated for protein-coding genes by PICRUSt and STAMP for investigating functional potential at levels 1, 2 and 3 (Table S8).

**Figure 4:**
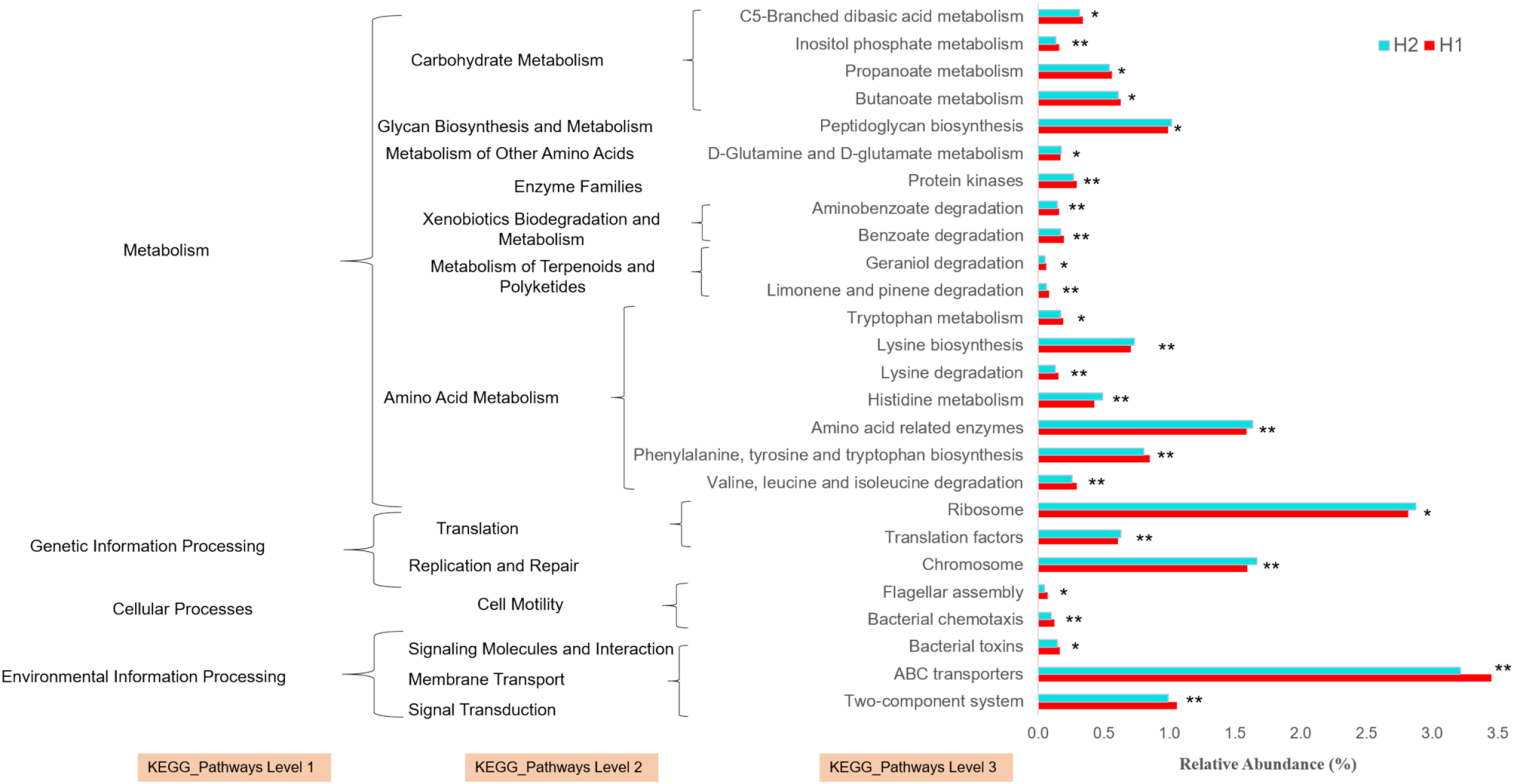
PICRUSt predicted Functional profile of Kyoto Encyclopedia of Genes and Genomes (KEGG) categories for oral microbiota with significantly different KEGG pathways at H1 and H2 using Welch’s t-test. Kyoto Encyclopedia of Genes and Genomes, (95% Confidence Interval (CI). Bar plots displayed the mean proportion of each KEGG pathway. P values were adjusted and corrected for the false discover rate. “*” and “**” indicate the significance level at 0.05 and 0.01, respectively.

Comparative analysis of microbial metabolic profiles at H1 and H2 demonstrate a significant decrease in functional genes, including two major metabolic pathways involving carbohydrates, and amino acids (Fig. 4). More specifically, in carbohydrate metabolism, butanoate (p < 0.05), propanoate (p < 0.05), inositol phosphate (p < 0.01), and C5-branched dibasic acid metabolism (p < 0.05) were majorly affected.

In amino acid metabolism, degradation of valine, leucine, isoleucine, and lysine, biosynthesis of phenylalanine, tyrosine, tryptophan, and lysine, amino acid related enzymes, metabolism of histidine, cyanoamino acid, and tryptophan, the urea cycle were significantly affected (Fig. 4). In addition to these primary essential metabolic pathways, other pathways affected were, metabolism of terpenoids and polyketides (e.g., limonene, pinene and geraniol degradation), xenobiotics biodegradation and metabolism (e.g., benzoate, and aminobenzoate degradation).

### Differential abundance analysis

**Figure 5:**
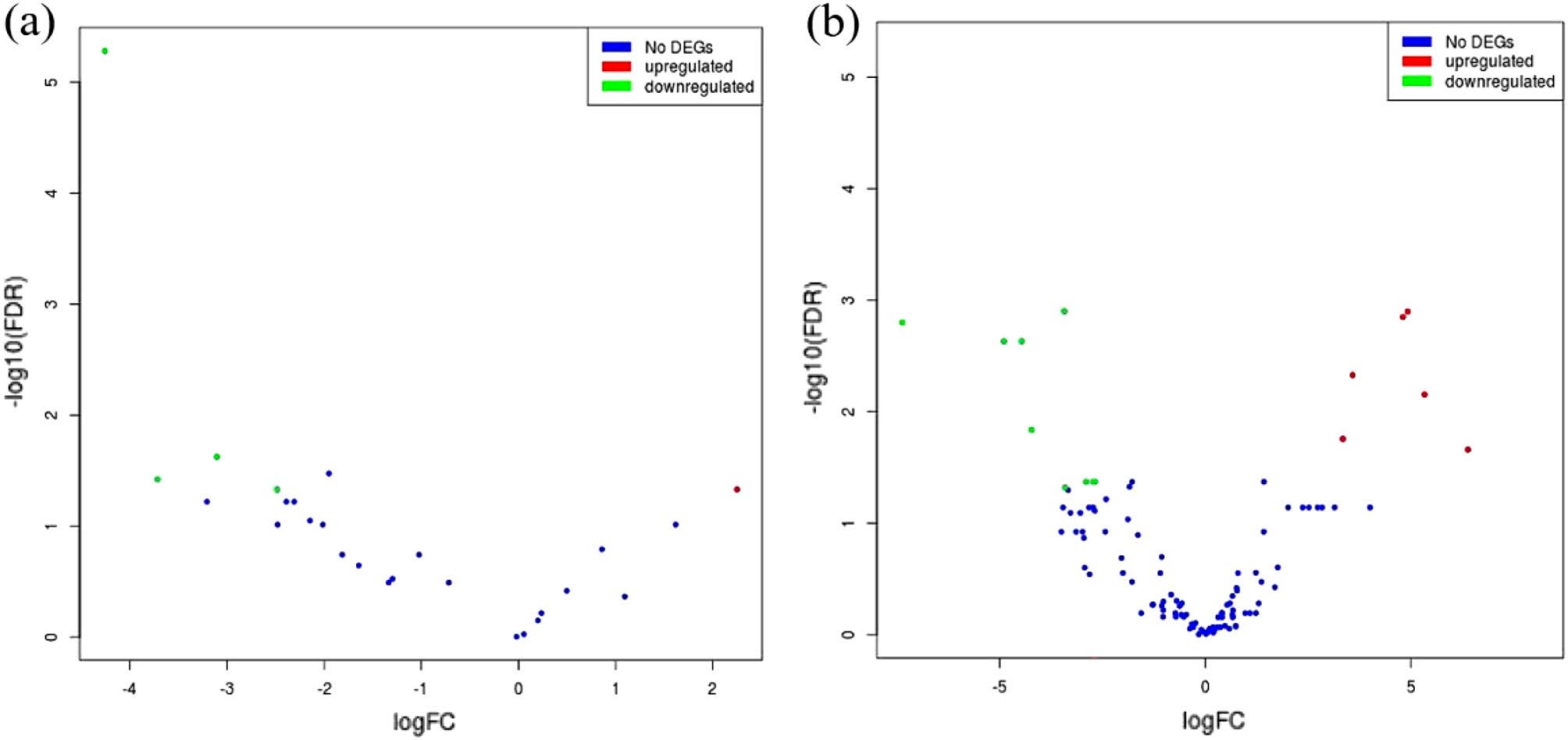
Volcano plot showing differential microbiota at (**a**) Phylum and (**b**) Genera level between H1 and H2 groups. Log-transformed fold change in expression is plotted on the x-axis and log-transformed false discovery rate-adjusted p-values plotted on the y-axis. The differential microbiota between the H1 and the H2 group were analyzed using the EdgeR package of the language R, in accordance with corrected P value < 0.05 and fold-change ≥  ± 2 and volcano plot was generated.

The analysis revealed 5 phylum and 16 genera differntially abundant between the two groups. Phylum namely, *Tenericutes* was highly abundant at H2 as compared to H1 while Phylum *TM7*, *Chloroflexi*, *Cyanobacteria*, and *Armatimonadetes* showed a lower abundance in the H2 group (Fig. 5a).

On the other hand, at genus level, 7 genera: *Pseudomonas, Comamonas, ML110J_20, Micrococcus, Gallibacterium, Hydrogenophaga*, and *Moraxella* showed a higher abundance at H2 group; and whereas 9 genera, namely, *Selenomonas, Peptoniphilus, Azoarcus, Acinetobacter, Paenibacillus, DA101, Rhodoplanes, Nocardioides*, and *Agrobacterium* showed a lower abundance in the H2 group (Fig. 5b).

### The potential link between taxonomy and functional pathways

We identified the correlation between several microbial genera with differential abundant functional pathways. Spearman’s rank correlation coefficient of microbial genera and predictive function pathways based on PICRUSt. The value r = 1 or close to 1, represents a strong positive correlation and the value r = − 1 or close to − 1, represents a strong negative correlation. p value < 0.05 was considered statistical significant.

Interestingly, the functional pathways of oral microbiota were found to have more and stronger correlations with microbial genera, (Tables 2 and S9). The majority of the pathways showed positive correlation (r = 0.086–0.621) with several groups of genera which implies that the influence of oral microbiota on the functional profile was more likely through the combinatorial effects of multiple bacteria, or microbial consortium, rather than individual microbial genera.

## Discussion

Microbes co-exist in and on the human body and greatly impact human health. The oral cavity which directly communicates with the external environment being portal of entry^29,30^, is one of the most significant factors impacting the oral microbiota^31^. Local oral environment and socio-environmental/economic variables have been ambiguous about the impact in on the makeup of the salivary microbiome which in turn reflects the integrity of periodontal health. In oral homeostasis, the microflora and host immunity have a symbiotic relationship^32^, as this balance promotes immunity in the oral cavity and enhances systemic immunity to prevent oral diseases^11^. However, when this balance is disrupted, it increases inflammation and may initiate several oral or systemic diseases^9,10,32,33^.

In the present pilot study, we characterized the salivary microbiome of 16 HA sojourners and evaluated variations caused by the environmental factors at HA. Such changes may lead to more dysbiosis in the oral microbiome, resulting aberrant inflammatory responses. Through the analysis, we found few significant changes in oral microbiota between the two heights. In terms of the composition of the oral microbiota, the abundance of *Firmicutes*, *Bacteriodetes*, and *Actinobacteria* decreased at H2. On the contrary, the abundance of *Proteobacteria* increased at H2 as compared to H1. Consistent with the results of other studies, a decreasing trend of *Firmicutes* with altitude (4500 m) was observed^21^. In addition, Fig. 1 shows that at phylum level, microbial communities at H2 were characterized by decreased abundance of *TM7* and increased abundance of *Tenericutes*, though their relative abundance was very low at sea level. At genera level, the abundance of *Streptococcus*, *Haemophilus*, *Actinomyces*, *Veillonella*, *Porphyromonas,* and *Granulicatella* decreased at H2 as compared with H1 and the abundance of *Neisseria*, *Rothia,* and *Fusobacterium* increased at H2. The cumulatively identified bacterial genera, were well represented in the human oral microbiome database and previously identified as members of the human salivary microbiome^2,34,35^. All of these genera are considered to be closely related to human health. For instance, *Porphyromonas* is not only associated with the occurrence of periodontitis, but it is also a driving factor for developing tumors in the gastrointestinal tract^36^.

In our study at genus level, the genera *Prevotella* occupies 8.9% at H1 and increased to 9.04% at H2. Though, it is a very minute and insignificant difference, but similar kind of pattern of increment with altitude has been reported by another study at 4500 m^21^. *Prevotella* is the predominant genus found in gut and oral microbiome at HA^42^. While the presence of *Streptococci* and *Prevotella* species is common in the general population, recent research has suggested that these bacteria may be associated with increased inflammation and other changes that could potentially contribute to the development of progression of oral cancer^37^. Reduction in salivary secretion at HA often gives a feeling of dry mouth and tongue^15^, and reduced salivary flow is directly correlated with altered oral microbiota composition^38,39^. Because the host is unable to balance the acidic environment, caries forming bacteria can flourish in this setting. In this study, we also report changes in bacterial genera including *Streptococcus, Actinomyces, Veillonella, Aggregatibacter*, and *Fusobacterium* which are engaged in the development and maturation of dental biofilms. However, it is important to note that the relationship between these bacteria and oral diseases at HA is not fully understood, and further research with a large sample size is needed to establish any causal links.

In addition, of these dominant genera in the oral cavity are not pathogenic in a healthy state, but they may cause diseases that affect the host’s health during compromised immune system and altered oral microbiota. HA has been known to cause alterations in different immune cells associated with innate and adaptive immunity. Mucosal immune system which is an important branch of adaptive immune response is also vulnerable to invading pathogens and infections at HA which further needs investigation^40,41^.

The temperature of the environment has an impact on the human microbiota, and thus, is a key determinant in bacterial abundance^42^. The ideal temperature of 37 °C, is considered a favorable temperature for growth of most parasitic microorganisms on the human body^43^. For example, compared to a German population living in a warmer climate, Alaskan humans living in a colder environment have much less alpha diversity of the oral microbiota^44^. In consistent with our study, a recent study also revealed that alpha diversity significantly decreased with increase in altitude^21^. Other studies have also shown that bacterial composition altered with the temperature drop^45,46^. Thus, it can be speculated that low temperature at HA is one of the contributing factors affecting the oral microbiome diversity (Table 1).

**Table 1:** Environmental features.

	H1 (Meerut)	H2 (Karakoram ranges)
Geographical location	28°57′ to 29°02′ N	32.89° to 33.12° N
	77°40′ to 77°45′ E	77.53°E to 77.78° E
Altitude	210 m	4420 m
Temperature during the stay period	10 °C (Lowest)	− 0.3 °C (Lowest)
	25 °C (Highest)	6.4 °C(Highest)

The functional prediction of oral bacterial populations from 16S marker sequences was elucidated by PICRUSt. The technique is frequently used, although it has certain drawbacks, including the requirement for OTUs as input sequences produced using closed-reference OTU-picking against the Greengenes database. Regarding microorganisms and their function in stepwise processes in a particular pathway, PICRUSt does not offer any clarity. However, adopting PICRUSt for functional prediction in this exploratory pilot study was done to establish the impact of altitude on functional potential of oral microbiome.

Decreased carbohydrate metabolism at H2 correlates with decreased abundance of saccharolytic genera *Streptococcus* and *Lactobacillus*. Another pathway, ABC transporters also showed decreased expression at H2. ABC transporter systems plays important biological roles in transport of oligosaccharides, including melibiose, raffinose, stachyose and maltodextran^47^ and its decreased activity leads to reduced carbohydrate metabolism and eventually reduced growth of saccharolytic bacteria. Butyrate and propionate are the most common SCFA (Short Chain Fatty Acids) after acetate. Reduced SCFAs level were reported to be responsible for increased oral mucosal TH_2_ immunity leading to risk of pro-inflammatory response in subjects with peanut allergy. This suggests that SCFAs can regulate the inflammatory response and may represent a link between the microbiota and the immune system^48,49^.

Furthermore, Xenobiotic biodegradation and metabolism correlates with the process of detoxification, where microbes play important role by degrading xenobiotics, which are usually caused by the release of industrial pollutants. In our findings, it is not surprising to find a decreased metabolic activity of xenobiotic biodegradation at HA, with minimal industrial pollution. We also emphasized on the relationship between genus and altered pathways using Spearman correlation (Table 2). PICRUSt datasets with 3^rd^ tier functional classification (Fig. 4) were employed to determine genus abundance contributing to altered functional pathways between the two heights. Interestingly, most of the genus showed a strong correlation with functional pathways, suggesting their involvement the functional traits. It was clearly evident genera such as *Pseudomonas* and *Acinetobacter* which were differentially abundant at H2 (Fig. 5b) were also found to be associated with metabolism pathways (Table 2). But at the same time genera consist of many species can negatively or positively regulate a pathway and show low taxonomical resolution in 16S rRNA sequencing reads is one of the limiting factors.

**Table 2:** Potential links between oral microbiota genera and functional pathways using Spearman’s rank correlation coefficient.

1st Tier	2nd Tier	3rd Tier	Spearman (r)	p value	Genus
Metabolism	Amino acid metabolism	Lysine biosynthesis	0.396	0.028	Campylobacter, Citrobacter, Enterobacter, Escherichia
	Amino acid metabolism	Lysine degradation	− 0.092	0.622	Citrobacter, Enterobacter, Escherichia, Fusobacterium
	Carbohydrate metabolism	Galactose metabolism	0.418	0.019	Citrobacter, Enterobacter, Escherichia, Fusobacterium
	Carbohydrate metabolism	Pentose phosphate pathway	0.446	0.012	Acinetobacter, Citrobacter, Enterobacter, Escherichia
	Energy metabolism	Nitrogen metabolism	0.086	0.645	Acinetobacter, Citrobacter, Enterobacter, Escherichia
	Energy metabolism	Sulfur metabolism	0.515	0.003	Citrobacter, Enterobacter, Escherichia, Neisseria
	Glycan biosynthesis and metabolism	Lipo-polysaccharide biosynthesis	0.133	0.475	Acinetobacter, Citrobacter, Enterobacter, Escherichia
	Glycan biosynthesis and metabolism	Peptidoglycan biosynthesis	0.451	0.011	Acinetobacter, Campylobacter, Citrobacter, Enterobacter
	Lipid metabolism	Fatty acid biosynthesis	0.621	0.000	Acinetobacter, Campylobacter, Citrobacter, Enterobacter
	Lipid metabolism	Glycero-phospholipid metabolism	0.176	0.342	Fusobacterium, Pseudomonas, Streptococcus
	Metabolism of cofactors and vitamins	Folate biosynthesis	0.361	0.046	Citrobacter, Enterobacter, Escherichia, Haemophilus
	Nucleotide metabolism	Purine metabolism	0.425	0.017	Acinetobacter, Campylobacter, Citrobacter, Enterobacter
	Nucleotide metabolism	Pyrimidine metabolism	0.447	0.012	Acinetobacter, Campylobacter, Citrobacter, Enterobacter
	Xenobiotics biodegradation and metabolism	Benzoate degradation	-0.170	0.362	Acinetobacter
	Xenobiotics biodegradation and metabolism	Toluene degradation	0.147	0.431	Pseudomonas
Genetic information processing	Replication and repair	DNA replication	0.274	0.136	Citrobacter, Escherichia, Haemophilus
	translation	Aminoacyl-tRNA biosynthesis	0.452	0.011	Acinetobacter, Campylobacter, Citrobacter, Enterobacter

The relationship between metabolic pathways and the genera responsible for has not been explored yet. Most of the pathways with significant alterations at 1, 2 and 3 tier are positively correlated to metagenome with r score more than 0. Some species differentially express pathways in association with other genera to produce active molecules, precursors, enzymes, hormones or metabolites to regulate host metabolism. However, dysbiosis may lead to alterations in the concerned metabolic pathways. Therefore, it needs an in depth analysis to fully elucidate the mechanistic interaction by which a group of genera participate in a metabolic pathway.

The results of this pilot study represents the exploratory description of the oral microbiome in individuals exposed to HA. Analysis of the salivary microbiome from the subjects residing at HA (4420 m above sea level) was compared with sea level controls for the first time and the findings of our study will provide preliminary baseline information for future research. Finally, our findings suggest that altitude has an impact on the oral microbiota's microbial composition, diversity, community structure, and function. Furthermore, future work should explore the relationship among altitude, oral microbiome, and periodontal health in large cohort is warranted to mitigate the problems encountered by the sojourners after travelling to or stay at HA.

## Limitations and merits

The main aim of this pilot study was to analyze the effects of extreme environmental conditions on the oral microbiota of subjects ascending to HA from sea level. However, some limitations were associated with the study such as small subject size which eventually limits the statistical power. But because of logistic issues, the availability of a small subject size is unavoidable. Additionally, the study was restricted to male participants exclusively. Due to the individuals' unavailability and unwillingness to provide follow-up samples after returning to 210 m, no follow-up analysis was feasible. Expanding the investigation of the oral microbiome using a combination of shotgun metagenomic, meta-transcriptomic, and metabolomics in a bigger cohort of population is warranted.

## Supplementary Information

Supplementary Tables.

Supplementary Figure S1.

## Data Availability

All the raw data obtained from 16S rRNA sequencing have been deposited to the NCBI sequence read archive (SRA) repository for the research community with the study accession number under Bioproject ID PRJNA836346.
