# Supplementary figures and images for: Impact of high altitude on composition and functional profiling of oral microbiome in Indian male population
Source: Sci Rep. 2023 Mar 10;13:4038. doi: 10.1038/s41598-023-30963-8 (PMC10006418; doi:10.1038/s41598-023-30963-8)

Supplementary **Fig. 1**

Rarefaction Curve


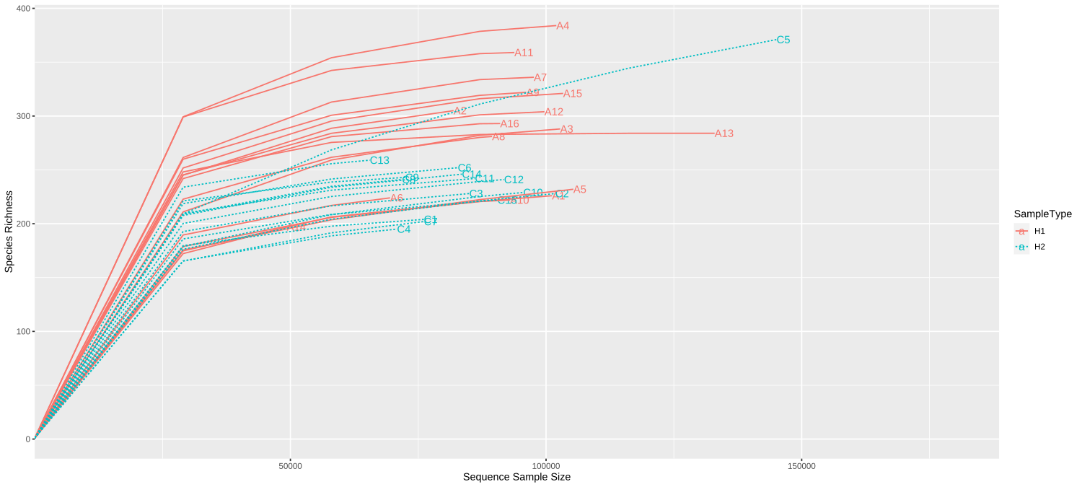


**Figure 1.**

Supplement: Supplementary file 2 — Supplementary Figure S1. [file 41598_2023_30963_MOESM2_ESM.docx]
